# Suicidal behaviour across the African continent: a review of the literature

**DOI:** 10.1186/1471-2458-14-606

**Published:** 2014-06-14

**Authors:** Becky Mars, Stephanie Burrows, Heidi Hjelmeland, David Gunnell

**Affiliations:** 1 School of Social and Community Medicine, University of Bristol, Oakfield House, Bristol BS8 2BN, United Kingdom; 2 Research Centre of the University of Montréal Hospital Centre, 3850 St-Urbain, H2W 1 T7 Montréal, Québec, Canada; 3 Department of Social and Preventive Medicine, University of Montréal, 7101 Avenue du Parc, H3N 1X7 Montréal, Québec, Canada; 4 Department of Social Work and Health Science, Norwegian University of Science and Technology, NO-7491 Trondheim, Norway

**Keywords:** Suicide, Suicide attempts, Africa, Review, Incidence, Risk factor, Sex, Method

## Abstract

**Background:**

Suicide is a major cause of premature mortality worldwide, but data on its epidemiology in Africa, the world’s second most populous continent, are limited.

**Methods:**

We systematically reviewed published literature on suicidal behaviour in African countries. We searched PubMed, Web of Knowledge, PsycINFO, African Index Medicus, Eastern Mediterranean Index Medicus and African Journals OnLine and carried out citation searches of key articles. We crudely estimated the incidence of suicide and suicide attempts in Africa based on country-specific data and compared these with published estimates. We also describe common features of suicide and suicide attempts across the studies, including information related to age, sex, methods used and risk factors.

**Results:**

Regional or national suicide incidence data were available for less than one third (16/53) of African countries containing approximately 60% of Africa’s population; suicide attempt data were available for <20% of countries (7/53). Crude estimates suggest there are over 34,000 (inter-quartile range 13,141 to 63,757) suicides per year in Africa, with an overall incidence rate of 3.2 per 100,000 population. The recent Global Burden of Disease (GBD) estimate of 49,558 deaths is somewhat higher, but falls within the inter-quartile range of our estimate. Suicide rates in men are typically at least three times higher than in women. The most frequently used methods of suicide are hanging and pesticide poisoning. Reported risk factors are similar for suicide and suicide attempts and include interpersonal difficulties, mental and physical health problems, socioeconomic problems and drug and alcohol use/abuse. Qualitative studies are needed to identify additional culturally relevant risk factors and to understand *how* risk factors may be connected to suicidal behaviour in different socio-cultural contexts.

**Conclusions:**

Our estimate is somewhat lower than GBD, but still clearly indicates suicidal behaviour is an important public health problem in Africa. More regional studies, in both urban and rural areas, are needed to more accurately estimate the burden of suicidal behaviour across the continent. Qualitative studies are required in addition to quantitative studies.

## Background

Each year, the World Health Organization (WHO) estimates that almost a million people die from suicide worldwide [1] highlighting suicide as a serious global public health concern. The contribution of suicide to the global burden of disease is predicted to increase over future decades [2]. Data from the WHO mortality database indicate that 85% of the world’s suicides occur in low and middle income countries (LAMIC) [3], however, most of our knowledge and understanding about suicidal behaviour is based on information from high income countries, which may not be applicable in different cultural contexts.

Africa is the world’s largest and second most populous continent, with a population of over one billion people. The continent is heterogeneous, comprising rural, semi-rural and urban areas, a diverse range of religions, ethnic groups and cultures and several regions affected by war, political and economic instability. Despite high overall mortality rates [1] suicide rates in Africa have been thought to be very low [4]. However, little is actually known about the incidence and patterns of suicide across the continent. This information is of fundamental importance, both to help inform local, regional and national policy, and to provide a more accurate estimate of the magnitude of suicide globally.

Suicide research in Africa is limited by a lack of systematic data collection. With less than 10% of African countries reporting mortality data to WHO, official statistics are available for only 15% of the continent’s total population. Much of the available published suicide data are based primarily on small studies conducted in different regions and populations. Moreover, reported suicide mortality statistics are likely to underestimate the true magnitude of the problem as religious and cultural sanctions may lead to suicide being under-reported, misclassified or deliberately concealed.

Even less is known about attempted suicide across the African continent. It is estimated that for every suicide that occurs worldwide there are up to 20 suicide attempts [5]; however reliable data are not available for most countries. Data are often obtained from hospital records which underestimate the number of cases, as many individuals are only admitted to hospital if in a critical condition. Moreover, a lack of access to medical facilities, particularly in rural areas of Africa, means that many suicide attempters are unlikely to present to hospitals. As with suicide, socio-cultural factors also contribute to under-reporting.

This paper reviews the published literature on suicide and suicide attempts in Africa to address these knowledge gaps. Specifically, we aim to:

i. Describe the incidence of suicide and suicide attempts across the African continent

ii. Describe common features across the studies, including information related to age, sex and methods used

iii. Identify key risk factors for suicide and suicide attempts.

## Methods

**Figure 1:**
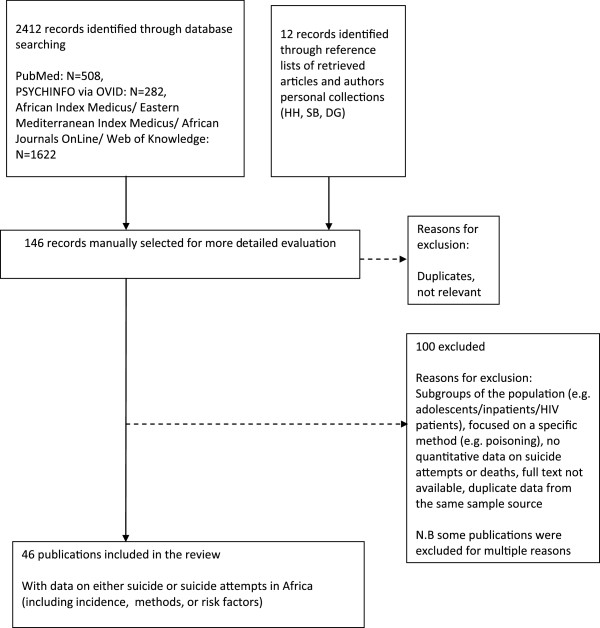
Flow diagram for data extraction.

A systematic search of PubMed, Web of Knowledge, PsycINFO, African Index Medicus, Eastern Mediterranean Index Medicus and African Journals OnLine was conducted for papers published between January 1998 and June 2013 that investigated fatal and non-fatal suicidal behaviour in an African country (incidence, methods or risk factors). Reference lists of relevant papers were examined for additional eligible studies and two of the authors (SB, HH), who have a longstanding interest in researching suicide in Africa, identified additional published publications from their personal collections of papers (Figure 1).

There has been much debate regarding the most suitable terminology to describe suicidal behaviour, a discussion of which is beyond the scope of this paper. In line with a newly proposed nomenclature for suicidal behaviour [6,7] we will use the term ‘suicide’ to refer to suicidal acts that result in death and ‘suicide attempts’ to refer to suicidal actions that do not result in death. Evidence of suicidal intent (whether explicit or implicit) is central to this definition, however, intention is often difficult to establish. It is possible that in some instances, acts of deliberate self-harm involving no suicidal intent may have been included.

The search was conducted using a combination of the following search terms: Suicide [Mesh] or suicid? [tw] or ideation [tw] or self harm? [tw] or self injur? [tw] and was restricted to articles published in English, German or French. The precise approach varied across the different databases. Papers were manually reviewed to assess eligibility for inclusion. In line with a public health perspective on the overall burden of suicide and suicide attempts across the continent, we focused on general population samples of adults. Case reports and publications focused on one sex or age group only (e.g. children and adolescents), or groups considered at high risk for suicide (e.g. patients attending psychiatric institutions, patients suffering from HIV/AIDS) were not included.

For those countries with more than one eligible publication (e.g. South Africa), the best available data are presented; these were selected according to several criteria including the recency of the publication, total population coverage and level of detail available. National-level suicide mortality data for the five countries that report data to WHO (Mauritius, the Seychelles, South Africa, Zimbabwe and Egypt) was obtained from the WHO mortality database [8].

### Deriving estimates of suicide and suicide attempts

If not reported in the publication, estimates of the annual incidence of suicide and suicide attempts per 100,000 population were derived wherever possible using the following calculation:

$$\frac{\mathrm{Number}\;\mathrm{of}\;\mathrm{suicides}/\mathrm{suicide}\;\mathrm{attempts}\;\mathrm{reported}\;\mathrm{per}\;\mathrm{year}}{\mathrm{Study}\;\mathrm{population}}\times 100,000$$

Where population data were not reported, estimates were obtained from on-line census data [9-11]; further details are provided in the table footnotes. Estimates are presented according to WHO mortality strata [1], which provide a way of describing the development of a country based on child and adult mortality rates. The mortality strata give a crude indication of the social and economic health of a country and allow countries at a similar stage of development to be grouped together. Thirty-eight percent of African countries are in highest mortality strata (Stratum E, high child and very-high adult mortality) and 58% in the second highest mortality strata (Stratum D, high child and high adult mortality). There were no data available for the 4% of countries in stratum B (Libyan Arab Jamahiriya and Tunisia).

The median annual suicide incidence rate was calculated within each stratum using the best available data for each country (Table 1). This estimate was then extrapolated to all countries within that stratum to give a rough estimate of the total number of deaths occurring each year.

$$\begin{array}{l} \frac{\mathrm{Total}\;\mathrm{population}\;\left[\mathrm{for}\;\mathrm{all}\;\mathrm{countries}\;\mathrm{within}\;a\;\mathrm{given}\;\mathrm{stratum}\right]}{100,000}\\ \times \;\mathrm{median}\;\mathrm{incidence}\;\mathrm{of}\;\mathrm{suicide}\;\mathrm{per}\;100,000\;\left[\mathrm{within}\;a\;\mathrm{given}\;\mathrm{stratum}\right] \end{array}$$

**Table 1:** Incidence of suicide in African countries with published data identified by our systematic search of the literature

**Country and source publication**	**WHO mortality stratum**	**Study population**	**Year**	**Mean annual suicide rates per 100,000 population**	**Sex ratio**
					**Male: female**
Cameroon					
Keugoung et al. [12]	D	Guidiguis health district	1999-2008	3.2^a^	3.7:1
Egypt					
Abdel Moneim et al. [13]	D	Assiut province	2005-2009	0.7^b^	1.4:1
Ghana					
Adinkrah [14]	D	National-level data	2006-2008	0.4^c^	21.1:1
Mauritius					
WHO [8]	D	National-level data	2008	6.8	6.0:1
Nigeria					
Nwosu & Odesanmi [15]	D	Ile-Ife	1979-1988	0.4	3.6:1
Senegal					
Guyavarch et al. [16]	D	Bandafassi, Niakhar and Mlomp	1985-2004	3.7	2.5:1
Seychelles					
WHO [8]	D	National-level data	2008	4.6	Males: 100%
Ethiopia					
Bekry [17]	E	Addis Ababa	1981/82-1995/96	7.8^d^	5.2:1
Kenya					
Ziraba et al. [18]	E	Viwandani and Korogocho slums in Nairobi City	2003-2005	3.3	Not specified
Malawi					
Dzamalala et al. [19]	E	Blantyre district	2000-2003	2.6	3.4:1
Mozambique					
Nizamo et al. [20]	E	Maputo City	2000	4.7	Not specified
Namibia					
Ikealumba & Couper [21]	E	Rehoboth	2001	2.3	Not specified
South Africa					
Burrows et al. [22]	E	Johannesburg, eThekwini, Cape Town, Tshwane, Nelson Mandela and Buffalo City	2001-2003	17.2	4.5:1
Uganda					
Kinyanda et al. [23]	E	Kampala city	1975-2004	1.0	3.4:1
Un. Rep. Tanzania					
Mgaya et al. [24]	E	Dar es Salaam region	2005	2.3	2.8:1
Zimbabwe					
WHO [8]	E	National-level data	1990	7.9	2.0:1

^a^average rate, range of annual rates 0.9-6.5 per 100,000; ^b^average rate, range of annual rates 0.6-0.8 per 100 000; ^c^average rate, range of annual rates 0.2-0.4 per 100,000; ^d^average rate, range of annual rates 3.3-11.7 per 100,000.

WHO: World Health Organization; data is presented for the most recent year available.

WHO mortality stratum D: high child mortality and high adult mortality; WHO mortality stratum E: high child mortality and very high adult mortality.

Five countries have additional publications available: South Africa [25-28], KwaZulu-Natal province, Transkei region, Pretoria and Bloemfontein city estimates range from 10.9 to 32.5 (average rate) per 100,000; Egypt [29], Port Said city average rate 2.2 per 100,000; Senegal [30], Dakar region 0.7 per 100,000; Uganda [31], Northern Uganda average rate 15.8 per 100,000; United Republic of Tanzania [32], Dar es Salaam 3.2 per 100,000.

South Africa and Egypt also have data available from mortality statistics they report to WHO [8].

Population estimated for the Dakar region, Senegal [30]. Source: National Agency of Statistics and Demography, Government of Senegal [9].

2012 population estimates for each country were sourced from WHO [33]; total population for countries in stratum D: 584,431,000 and stratum E: 469,722,000. Values were also calculated for the lower and upper quartile in order to illustrate the level of uncertainty within the estimate. Estimates across strata were then combined to provide a crude estimate of the total number of suicide deaths occurring in Africa each year. The figures presented are intended to provide only a rough estimate and need to be interpreted with great caution given the lack of suicide data for many countries, the variability in estimates both within and across countries, the lack of national-level suicide data and the likely under-reporting of suicide.

The recent Global Burden of Disease (GBD 2010) study [34] has produced estimates of country-specific suicide rates for Africa. We compared these estimates with those derived from our review of the published literature. The GBD study did not evaluate the incidence of attempted suicide, risk factors for suicide nor commonly used methods of suicide.

## Results

### Incidence

#### i. Suicide

**Figure 2:**
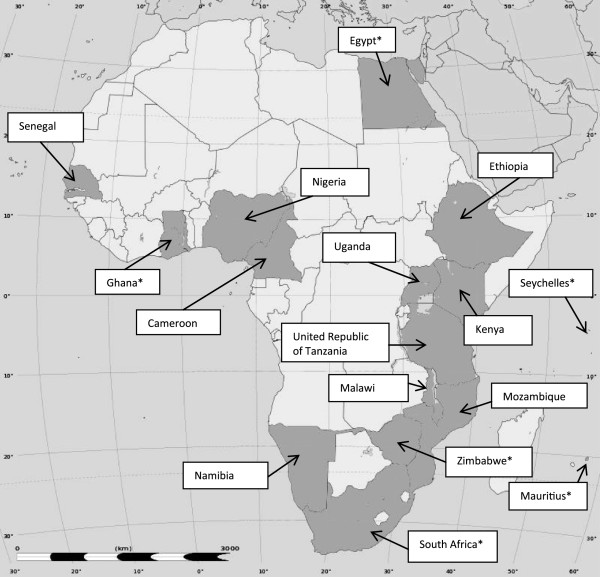
Countries in Africa with suicide incidence data available. Source: Wikimedia Commons, courtesy of Eric Gaba. National-level suicide incidence data are lacking for many African countries. *Countries for which national-level suicide incidence data are available.

The best available data for the incidence of suicide in each country are presented in Table 1. Data are available for 16 African countries which together account for approximately 60% of the total population of Africa; although frequently data are available for only a small proportion of the population within a country (Figure 2). The median incidence of suicide for the nine countries in the highest mortality stratum (E) was 3.3 [inter-quartile range 2.3 to 7.9] per 100,000 and for the seven countries in the second highest stratum (D) was 3.2 [inter-quartile range 0.4 to 4.6] per 100,000. Based on these data, we crudely estimate the annual number of suicides in Africa to be approximately 34,000 [inter-quartile range 13,141 to 63,757]; this is based on an estimated ~19,000 suicides in stratum D countries and ~15,000 suicides in stratum E countries. The overall annual incidence rate is estimated to be 3.2 per 100,000. This figure compares with an estimate of 49,558 suicides (median incidence rate 4.8 per 100,000) derived from the recent GBD study 2010 [34]. A country-level comparison of suicide incidence rates estimated in the GBD study and the best available data from our literature review is presented in Table 2. Literature-based estimates for specific countries were most discrepant from the GBD estimates for South Africa (higher), Zimbabwe (lower), Uganda (lower), Malawi (lower) and Tanzania (lower).

**Table 2:** Comparison of global burden of disease estimates with published data identified by our systematic search of the literature

**Country**	**Global burden of disease estimate:**	**Published data estimate:**
	**Mean annual suicide rates per 100,000 population (2010)**	**Mean annual suicide rates per 100,000 population**
Algeria	1.4	
Angola	5.0	
Benin	2.1	
Botswana	3.3	
Burkina Faso	1.7	
Burundi	10.8	
Cameroon	3.0	3.2^a^
Cape Verde	4.3	
Central African Republic	9.9	
Chad	1.7	
Comoros	8.1	
Congo	7.0	
Côte d’Ivoire	3.2	
Democratic Republic of the Congo	4.6	
Djibouti	8.3	
Egypt	2.0	0.7^b^
Equatorial Guinea	6.4	
Eritrea	8.3	
Ethiopia	7.5	7.8^c^
Gabon	9.8	
Ghana	1.5	0.4^d^
Guinea	1.7	
Guinea-Bissau	2.1	
Kenya	7.0	3.3
Lesotho	7.0	
Liberia	2.0	
Libya	2.8	
Madagascar	6.3	
Malawi	11.4	2.6
Maldives^e^	6.9	-
Mali	1.6	
Mauritania	1.8	
Mauritius	7.1	6.8
Morocco	3.4	
Mozambique	12.1	4.7
Namibia	5.0	2.3
Niger	1.3	
Nigeria	1.9	0.4
Rwanda	6.3	
São Tomé and Príncipe	1.6	
Senegal	2.2	3.7
Seychelles	11.8	4.6
Sierra Leone	2.0	
Somalia	6.7	
South Africa	3.6	17.2
Sudan	7.6	
Swaziland	8.6	
Tanzania	9.3	2.3
The Gambia	1.8	
Togo	2.3	
Tunisia	2.3	
Uganda	6.9	1.0
Zambia	8.8	
Zimbabwe	26.8	7.9
**Estimated total suicide deaths**	**49,558**	**34,203**

^a^average rate, range of annual rates 0.9-6.5 per 100,000; ^b^average rate, range of annual rates 0.6-0.8 per 100 000; ^c^average rate, range of annual rates 3.3-11.7 per 100,000; ^d^average rate, range 0.2-0.4 per 100,000 ^e^Publications from the Maldives were not included in our literature search.

Several countries have data available from multiple publications including five for South Africa (mean annual estimates range from 10.9 to 32.5 per 100,000 population) [22,25-28] and two for Egypt (mean annual estimates 0.7 and 2.2 per 100,000 population) [13,29], Senegal (mean annual estimates 0.7 and 3.7 per 100,000 population) [16,30], Uganda (mean annual estimates 1.0 and 15.8 per 100,000 population) [23,31] and the United Republic of Tanzania (mean annual estimates 2.3 and 3.2 per 100,000 population) [24,32]. In addition, South Africa and Egypt have WHO mortality data available [8], and for both countries WHO rates are considerably lower than in the publication (South Africa 0.9 vs. 17.2 per 100,000 [22]; Egypt 0.1 vs. 0.7 [13]). Additional studies from South Africa [25-28] and Egypt [29] report similarly high rates, indicating that WHO data are likely to be underestimated.

##### Secular trends

Data on secular trends in suicides are unavailable for many African countries. Six countries report suicide incidence data for five or more time points, including three countries from the WHO mortality database (Mauritius, Seychelles and Egypt). The trend for Mauritius (12 time points; 1955–2008) shows an initial decline in suicide rates followed by a substantial increase; rates increased from 1.7 in 1970 to 14.1 in 1990, after which rates have been steadily declining. Data from the Seychelles (5 time points; 1985–2008) show initially fluctuating rates which have been declining since 1998. Suicide incidence rates in Egypt appear to be relatively stable over time (5 time points; 1974–2009).

Trend data from peer reviewed publications are available for four countries. Similar to the findings from WHO statistics, data for Egypt show little variability in rates over time [13]. Trends in Cameroon (annual suicide incidence rates over 10 years;1999 to 2008 [12]) show a general increase in rates over time, with two notable decreases between 2001–2003 and 2006–2007. Rates increased substantially between 2003 and 2006 from ~1.5 to ~6.5 per 100,000 population. Rates in Ethiopia (annual suicide incidence rates reported over the 15 year period 1981/2 to 1995/6 [17]) vary substantially across time. Trend data are available for South Africa from multiple publications; one study shows substantial fluctuations in rates over time (2000–2007) [25], one minor fluctuations (2002–2008) [35], and one an initial decline in suicide rates followed by an increase (1996–2000) [28]. There is also some evidence to suggest that secular trends may be influenced by demographic factors such as age and race [36].

##### Sensitivity analyses

There is wide variability across countries regarding the time period over which suicide data were collected (range 1975–2009). In order to examine the impact of this on our estimates, sensitivity analyses were conducted excluding those countries where the majority of the data were collected prior to 2000 (Nigeria, Ethiopia, Senegal, Uganda and Zimbabwe). The median estimates for each stratum remained largely unchanged (stratum D: 3.2 and stratum E: 3.0).

We also conducted sensitivity analyses to examine the extent to which our estimate of the annual number of suicides in Africa differs when calculated using the mean as opposed to the median suicide incidence rate for each strata. When using the mean, the estimated number of suicide deaths was 42,199 (approximately 20% higher than our estimate of 34,000), but remained below the GBD estimate of 49,558.

#### ii. Suicide attempts

Data are available on suicide attempts for 11 countries, seven of which report rates per 100,000 (Table 3). Incidence rates vary widely from 0.1 per 100,000 in Ghana [14] to 100 per 100,000 in Namibia [21]. The remaining four studies report lifetime prevalence estimates for suicide attempts collected primarily from surveys. Estimates vary from 0.7% in Nigeria [37] to 6.0% in Liberia [38]. Two countries have data available from more than one publication (South Africa estimates range from 2.9%-3.4% [39,40] and Ethiopia estimates range from 0.9%-3.2% [41,42]). A summary of the best available data (range, median and mean) for both suicide and suicide attempts according to WHO mortality strata is presented in Table 4.

**Table 3:** Incidence of suicide attempts in African countries with published data identified by our systematic search of the literature

**Country and source publication**	**WHO mortality stratum**	**Study population**	**Year**	**Mean annual suicide attempt rates per 100,000 population/Lifetime prevalence**	**Sex ratio**
					**Male:Female**
Ghana					
Adinkrah [14]	D	National-level data	2006-2008	0.1 per 100,000^a^	10:1
Liberia					
Johnson et al. [38]	D	Sampled from whole country [n = 1,666]	May 2008	Lifetime prevalence: 6.0%	0.7:1
Morocco					
Agoub et al. [43]	D	Casablanca, random sample [n = 800]	Not available	Lifetime prevalence: 2.1%	0.5:1
Nigeria					
Gureje et al. [37]	D	Sampled from 5 of the 6 geopolitical regions [n = 6752]	Feb 2002-May 2003	Lifetime prevalence: 0.7%	1.0:1
Ethiopia					
Bekry [17]	E	Addis Ababa (hospital presentations)	1981/82-1995/96	49.8 per 100,000^b^	2.9:1
Malawi					
Dzamalala et al. [19]	E	Blantyre district (hospital presentations)	2000-2003	10.7 per 100,000	0.8:1
Namibia					
Ikealumba & Couper [21]	E	Rehoboth (hospital presentations)	2001	100.0 per 100,000	0.9:1^c^
South Africa					
Joe et al. [39]	E	Sampled from whole country [n = 4,351]	Jan 2002-June 2004	Lifetime prevalence: 2.9%	0.3:1
Uganda					
Kinyanda et al. [44]	E	Kampala (hospital presentations)	Jan 2002-Oct 2002	10.1 per 100,000	1.7:1
Un. Rep. Tanzania					
Ndosi & Waziri [45]	E	Dar es Salaam (hospital presentations)	Jan 1991-June 1993	5.2 per 100,000	0.5:1
Zimbabwe^d^					
Chibanda et al. [46]	E	Harare (hospital presentations)	Jul 1997-Dec 1997	49.9 per 100,000	0.2:1

^a^average rate, range of annual suicide attempts 0.03 to 0.08 per 100,000; ^b^average rate, range of annual suicide attempts 7.4 to 163.0 per 100,000; ^c^sex rates for suicide attempts include one suicide; ^d^Incidence figures do not include those admitted to the intensive care unit.

WHO: World Health Organization.

WHO mortality stratum D: high child mortality and high adult mortality; WHO mortality stratum E: high child mortality and very high adult mortality.

Two countries have additional publications available: South Africa [40], Durban lifetime suicide attempt rate 3.4%; Ethiopia [41,42], Addis Ababa and Butajira lifetime suicide attempt rates 0.9% and 3.2%.

Population estimated for Harare, Zimbabwe [46] based on data from the 1992 and 2002 Zimbabwe census. Source: Central Statistical Office [10,11].

**Table 4:** Summary of data for suicide and suicide attempts in Africa according to mortality strata

	**Range**	**Median (lower and upper quartile)**	**Mean**
**WHO mortality stratum D**			
**(58% of African countries)**			
**Suicide**			
7 countries; rate per 100,000	0.4 - 6.8 per 100,000	3.2 per 100,000 (0.4, 4.6)	2.8 per 100,000
**Suicide attempts**			
3 countries; lifetime prevalence	0.7% - 6.0%	2.1% (1.4, 4.1)	2.9%
1 country; rate per 100,000	0.1 per 100,000	0.1 per 100,000	0.1 per 100,000
**WHO mortality stratum E**			
**(38% of African countries)**			
**Suicide**			
9 countries; rate per 100,000	1.0 - 17.2 per 100,000	3.3 per 100,000 (2.3, 7.9)	5.5 per 100,000
**Suicide attempts**			
1 country; lifetime prevalence	2.9%	2.9%	2.9%
6 countries; rate per 100,000	5.2 - 100 per 100,000	30.3 per 100,000 (10.1, 49.9)	37.6 per 100,000

WHO: World Health Organization.

WHO mortality stratum D: high child mortality and high adult mortality; stratum E: high child mortality and very high adult mortality.

No data were available for the 4% of African countries in stratum B.

Number of annual deaths = population of countries in stratum/100,000 X median incidence rate for stratum.

Estimated number of annual deaths in stratum D countries = 584,431,000/100,000 X 3.2 = 18,702.

Estimated number of annual deaths in stratum E countries = 469,722,000/100,000 X 3.3 = 15,501.

Estimated number of annual deaths in Africa: 34,203.

### Sex and age differences

#### i. Sex

Data on sex differences in the incidence of suicide are available for 13 countries (Table 1). All studies reported higher rates in males with most reporting a male to female ratio of at least 3:1.

Evidence for sex differences in suicide attempts is less clear. Data are available for 11 countries (Table 3), of which five studies report a clear female predominance [38,39,43,45,46], three studies find a clear male predominance [14,17,44] and three studies report similar rates for males and females [19,21,37].

#### ii. Age

Age-specific rates were available from WHO for five countries [8]. The lowest rates were generally found in those under the age of 25 with few suicides reported in children under 15 years (≤0.5 per 100,000). There was little variability in age-specific rates for Egypt (0.0-0.2 per 100,000). Suicide rates in the Seychelles increased with age until 55 years, after which there were no reported suicides. In Mauritius and Zimbabwe, rates were highest amongst older adults (aged 55+). Rates in South Africa were highest in those 15–54 and those over 75.

Only one publication reported age-specific rates of suicide per 100,000. In the United Republic of Tanzania [24] rates were highest amongst those aged 45–59 years (5.7 per 100,000) followed by those aged 30–44 years (4.0 per 100,000). Thirteen publications from eight countries reported either the mean or median age at suicide or the proportion within specific age bands [12,13,15,19,23,26,27,29-32,47,48], however these estimates should be interpreted with great caution as they are influenced by the age distribution of the general population. Mortality records are also often incomplete.

For suicide attempts, age information was reported in eleven publications from seven countries [17,21,37,39,41,42,44-46,49,50]. These studies consistently showed highest rates amongst young adults (aged 15–30 years).

### Methods used

Nineteen publications from ten countries reported methods used for suicide [12,14,15,17,19,22-27,29-32,35,48,51,52]. The best available data for each country are presented in Table 5. The predominant methods for suicide were hanging and poisoning, although rates varied considerably across studies (hanging 8% - 70%; poisoning 8% - 83%). Firearms were also a common method in some countries (range 0% - 32%). Further details about the poisonous toxins used were reported in eight countries. In Cameroon, Egypt, Malawi, Nigeria and Uganda most poisoning deaths were attributable to pesticides (typically organophosphorous, organochlorine and rodenticides) with low rates of medication overdose [12,15,19,23,29]. In comparison, studies from Senegal and the United Republic of Tanzania report higher rates of medicine overdose [30,32], whilst similar rates of medicine overdose and pesticide poisoning were found in South Africa [27].

**Table 5:** Predominant suicide methods in African countries with published data identified by our systematic search of the literature

**Country and source publication**	**Study population**	**Predominant method(s)**
Cameroon		
Keugoung et al. [12]	Guidiguis health district	Poisoning (83%; 77% agricultural chemicals, 6% non-agricultural chemicals)
		Hanging (17%)
Egypt		
Gad ElHak et al. [29]	Port Said city	Poisoning (34%; Rodenticides 25%, medication [barbiturates and opiates] 9%)
		Drowning (19%)
		Burning (16%)
		Firearms (14%)
		Jumping (10%)
		Hanging (8%)
Ethiopia		
Bekry [17]	Addis Ababa	Hanging (70%)
		Drowning (15%)
		Poisoning (8%)
Ghana		
Adinkrah [14]	National-level data	Hanging (61%)
		Firearms (17%)
		Poisoning (11%)
Malawi		
Dzamalala et al. [19]	Blantyre district	Poisoning (79%; Temik [carbamate] and organophosphate)
		Hanging (19%)
Nigeria		
Nwosu & Odesanmi [15]	Ile-Ife	Firearms (32%)
		Hanging (20%)
		Poisoning (37%; of which 86% Gammalin [organochlorine])
		Cutting (5%)
Senegal		
Soumah et al. [30]	Dakar region	Hanging (44%)
		Poisoning (37%; mostly medications [Chloroquine] and organochlorines)
		Firearms (6%)
		Cutting (5%)
South Africa		
Stark et al. [27]	Bloemfontein	Hanging (56%)
		Firearms (21%)
		Poisoning (16%; medications 9%)
Uganda		
Kinyanda et al. [23]	Kampala city	Hanging (63%)
		Poisoning (26%; Mostly organophosphates; medications 0.3%)
		Jumping (5%)
		Firearms (5%)
Un. Rep. Tanzania		
Ndosi et al. [32]	Dar es Salaam region	Poisoning (69%; 28% antimalarials [mostly Choroquine]); 12% pesticide [Steladone/Diazinone [organophosphates]) and 29% could not be identified)
		Hanging (27%)

Methods ≥5% shown.

Five countries have additional publications available: South Africa [22,25,26,35,51]; Nigeria [48]; Ethiopia [52]; Uganda [31] and the United Republic of Tanzania [24].

Nine publications from seven countries reported methods used for suicide attempts [14,21,41,42,44-46,49,50]. The predominant method was poisoning which was reported in 26% [42] to 91% [45,49] of attempts. Medicine overdose was more common than pesticide poisoning in Namibia, South Africa, Zimbabwe and the United Republic of Tanzania [21,45,46,49], whereas pesticides were marginally more common in Uganda [44]. The most frequently used medications were antidepressants, antimalarials and psychotropic medications. Cutting was a common method in two studies [14,21] and hanging in three studies [14,41,42].

Results stratified by sex showed some sex differences in methods [12,15,19,24,26,29,30,32,41,42,44,48,50]. For both suicides and suicide attempts females generally had higher rates of poisoning than males, whereas males were more likely than females to use violent methods such as hanging and firearms.

### Risk factors

#### i. Suicide

Nine publications from four countries reported risk factors for suicide [12,23,24,26,31,32,47,51,53], often collected via psychological autopsies with relatives. Mental health problems were reported to play a role in up to 11% of suicides [12,32]. Physical health problems were also reported [12,24,32,51], for example in the United Republic of Tanzania, the rate of HIV was double amongst those who died by suicide when compared to the national prevalence for sexually active adults [32]. Alcohol and/or drug use was a prominent risk factor [12,24,26,31,32,47,51,53], with one study reporting that alcohol was involved either directly or indirectly (via the drinking behaviour of significant others) in as many as 80% of suicides [53]. Studies in South Africa also found that approximately 40% of individuals who died by suicide tested positive for alcohol on blood assays [26,47]. Interpersonal and social difficulties including family conflict, friendship or relationship problems and unwanted pregnancy [12,23,24,31,32,51], and socioeconomic factors [12,23,24,31,32,51] also play an important role.

The only case control study to investigate precipitating factors for suicide [23] found higher levels of psychological distress amongst individuals who died by suicide relative to road traffic collision victims, however, no differences were found in mental health problems, physical health problems, family conflict or drug/alcohol abuse.

#### ii. Suicide attempts

Ten publications from seven countries reported risk factors for attempted suicide [21,37,41,44-46,49,54-56], most often via population surveys or interviews with patients/relatives. As for suicide, commonly identified risk factors included interpersonal and social difficulties [21,41,44-46,49], physical illness [41,44,45,49] and socioeconomic factors [41,44,45,49]. Mental health problems were a prominent risk factor [37,41,44,45,49,54] with one study finding a four-fold increase in odds of suicide attempt amongst those with psychiatric disorder [54]. The risk of suicide attempt was considerably higher amongst those with multiple disorders [37,54]. Alcohol/drug use was also reported as a risk factor [41,44,45,49], with men more likely to report substance use than women [44,49]. Several other risk factors were identified including elevated numbers of negative life events, feelings of guilt or shame, sexual problems, feelings of loneliness, poor self-esteem, childhood abuse/trauma, parent mental health problems and family suicidal behaviour [37,44,45,49,55,56].

### Qualitative research

To understand how risk factors may be connected to suicidal behaviour, qualitative studies are needed which are able to take more of the complexity of suicidal behaviour and the socio-cultural context into consideration [57,58]. To our knowledge, only two qualitative studies on suicidal behaviour have been conducted in Africa to date; one on suicide and one on attempted suicide.

In post-conflict Northern Uganda, Kizza et al. [59,60] conducted a qualitative psychological autopsy study among men and women in Internally Displaced Peoples’ camps. In this context, suicide in both sexes was found to be connected to men’s “loss of masculinity”. In order to understand what this means it is necessary to understand how the gender roles in this community had been before the war and how the war had changed these roles and responsibilities in a way that contributed to suicide for both men and women, albeit in very different ways [59,60].

Quantitative studies on suicidal behaviour have commonly found religion/religiosity to be a protective factor. However, in a qualitative interview study with suicide attempters in Ghana, Akotia et al. [61] found that that was not necessarily the case. For instance, some individuals had attempted suicide because they were disappointed with God; they had fulfilled their religious obligations and did therefore not understand why God still allowed suffering in their lives.

## Discussion

Knowledge about suicide in Africa is limited with less than 10% of countries reporting mortality data to WHO. This investigation aimed to increase our understanding of the prevalence, patterns and risk factors for suicide and attempted suicide across the continent by reviewing and consolidating the available literature.

Suicide incidence rates have been reported in only 16 countries which together account for approximately 60% of the total population of Africa. However, national-level suicide data are lacking for most of these countries. There was considerable variation in the rates reported, both within and across countries. This could reflect the unreliability of the data, or alternatively could highlight the importance of the cultural context. Suicide rates in urban South Africa are reported to be much higher than in the other countries [22], perhaps due to the better quality and reliability of mortality data available.

Based on the limited available data, we crudely estimated the annual number of suicides in Africa to be approximately 34,000 [inter-quartile range 13,141 to 63,757]. This figure was calculated from median incidence rates [according to mortality strata] and is intended to provide only an approximation to the incidence of suicide in Africa. This estimate is somewhat lower than the recent GBD 2010 estimate of 49,558 deaths [34], however our figure is based only on available published data and does not take into account the geographical, sex or age structure of countries. It is not possible to judge which estimate is more accurate as each is based on different sources and assumptions. Any current estimates of suicide in Africa need to be interpreted with great caution given the absence of data for many countries, the variability of estimates and the lack of national-level statistics. Moreover, the huge cultural and religious diversity found both within and across African countries together with geographic (i.e. rural/urban), economic and political differences mean results based on data from one population or region are unlikely to be generalisable to another. Additional research is urgently needed, particularly in rural and economically deprived regions where suicide data are largely absent. Studies from India, China and Sri Lanka indicate that rural areas have exceptionally high rates of suicide [62-64], most likely due to easy access to pesticides combined with poor access to medical facilities and ineffective treatments.

The lifetime prevalence of suicide attempts also varied across studies, with a median estimate of 2%-3% [range 0.7-6.0%]. This compares with 0.4-4.2% found in the WHO SUPRE-MISS community survey of LAMICs [40] and 2.7% from the 17 countries in the World Mental Health Surveys [65]. Worldwide it is estimated that there are up to 20 suicide attempts for every suicide death [5] but across much of the African continent, the ratio of deaths to attempts appears to be much lower than this. Whilst this ratio does vary globally by country and suicide method, it could indicate that the true incidence of suicide in Africa is underestimated. In many African countries, suicidal behaviour carries negative religious and cultural sanctions and therefore may be under-reported, hidden or deliberately misclassified. In addition, the uncertainty in establishing suicidal intent may lead some suicidal acts to be misclassified as unintentional. There is some evidence from South Africa to suggest that suicide deaths by poisoning, jumping and railways are more likely to be misclassified than those by firearms or hanging [66].

Worldwide, three to four more men die by suicide than women. The ratio is much lower in Asian countries [67,68] and in China, more women die from suicide than men, particularly in rural areas [62,63,68]. Available evidence from Africa suggests that sex differences in suicide are broadly consistent with international trends, with all countries reporting a male predominance, typically at a ratio of 3.0:1 or higher. However, most studies have been conducted in urban areas and it is not clear whether this pattern would also be seen in rural areas of Africa. The sex discrepancy identified in this review may in part be explained by a propensity for men to use more lethal methods such as hanging and firearms, whereas the most common method used by women was poisoning.

Clear conclusions cannot be made regarding sex differences in attempted suicide in Africa as some studies reported a male predominance, some a female predominance, and others no clear sex differences. These findings contrast with international trends where suicide attempts tend to be 2–3 times higher in women than in men [50,69].

Knowledge of the most prominent methods used for suicide in Africa is vital for the development of prevention strategies, as restricting access can be an effective way of reducing suicide rates [2,70,71]. According to WHO, pesticide poisoning is now the most common method of suicide worldwide [72] and is frequently reported in China, Sri Lanka and India, particularly in rural areas [64]. Findings from this review suggest that pesticide poisoning is also a prominent method in Africa. Moreover, the proportion of suicidal acts involving pesticides is likely to be underestimated as data are largely absent in rural areas where pesticides are easily accessible and likely to be a commonly used suicide method. Evidence from Sri Lanka [73,74] suggests that reducing access to pesticides by banning those that are most toxic to humans is an effective means of reducing suicide rates. Improved medical management for pesticide poisoning is also urgently needed in order to reduce the lethality of this method. Poisoning with over-the-counter medications was also a common method used in both suicide and suicide attempts. Legislation regarding quantities of over-the counter medication may help to reduce overdose rates [75].

Given their high lethality, it is not surprising that rates of hanging and firearms were higher for suicide than for suicide attempts. The use of firearms as a method for suicide varied considerably across studies [range 0-32%], probably reflecting differences in the availability of this method. Prevention approaches should focus on restricting access to firearms and promoting safer storage [70,71].

A greater understanding of the antecedents to suicide is important in order to identify high-risk groups and to develop effective prevention strategies. Suicide is multi-factorial, involving a complex interplay of biological, social, cultural and psychological factors. Information about suicide risk factors in Africa is typically obtained retrospectively, either from medical records which are often incomplete, or from relatives’ reports which may be biased. Case control and cohort studies are required to better characterise risk factors for suicidal behaviour in Africa. For example, several studies reported high rates of unemployment amongst those who had died by suicide; however, as rates of unemployment are generally high within the population, the absence of a suitable comparison group means that these data are not informative.

Risk factors for suicide and suicide attempts identified in this review include physical health problems, psychiatric disorder or symptoms, drug and alcohol use/abuse, interpersonal and social difficulties and socioeconomic problems. The type of risk factors that are identified and their relative importance is likely to vary across different regions and population groups. The importance of taking into consideration the socio-cultural context has been highlighted by qualitative studies [59-61]. Such studies are crucial in order to build locally relevant suicide theory and to understand how, when, where and for whom risk factors may be connected to suicidal behaviour [57,58].

## Conclusions

Knowledge about suicide and suicide attempts in Africa is important, not only for African policy but also to improve the precision of global estimates of the magnitude of suicide. Findings from this review suggest that suicide is an important public health issue in Africa, with reported figures highly likely to underestimate the true incidence. Systematic data collection is urgently required in order to compile reliable suicide mortality and morbidity statistics across the continent. There is also a need for more qualitative studies, which are able to take into account the socio-cultural context.

## Abbreviations

WHO: World Health Organization; LAMIC: Low and middle income countries; GBD study: Global Burden of Disease study.

## Competing interests

The authors declare that they have no competing interests.

## Authors’ contributions

DG and BM contributed towards the conception of the study. BM helped to review and consolidate the literature, searched reference lists of eligible articles, assisted with the interpretation of the data and drafted the manuscript. SB, HH and DG assisted with the acquisition of relevant articles/data, participated in the interpretation of the data, assisted with the drafting of the manuscript and critically appraised the manuscript for important intellectual content. All authors read and approved the final manuscript.

## Pre-publication history

The pre-publication history for this paper can be accessed here:

http://www.biomedcentral.com/1471-2458/14/606/prepub
